# Metastatic Clear-Cell Renal Cell Carcinoma in the Era of Immune Checkpoint Inhibitors: Therapies and Ongoing Trials

**DOI:** 10.3390/cancers14122867

**Published:** 2022-06-10

**Authors:** Tony Zibo Zhuang, Katherine Case, Timothy Anders Olsen, Jacqueline T. Brown, Bradley C. Carthon, Omer Kucuk, Jamie Goldman, Wayne Harris, Mehmet Asim Bilen, Bassel Nazha

**Affiliations:** 1 School of Medicine, Emory University, Atlanta, GA 30322, USA; ztzhuan@emory.edu (T.Z.Z.); katherine.case@emory.edu (K.C.); timothy.a.olsen@emory.edu (T.A.O.); 2 Winship Cancer Institute, Emory University, Atlanta, GA 30322, USA; jacqueline.theresa.brown@emory.edu (J.T.B.); bradley.c.carthon@emory.edu (B.C.C.); omer.kucuk@emory.edu (O.K.); jamie.m.goldman@emory.edu (J.G.); wharr01@emory.edu (W.H.); mehmet.a.bilen@emory.edu (M.A.B.); 3 Department of Hematology and Medical Oncology, Emory University School of Medicine, Atlanta, GA 30322, USA

**Keywords:** clear-cell renal cell carcinoma, immunotherapy, immune checkpoint inhibitor, nivolumab, ipilimumab, pembrolizumab, tyrosine kinase inhibitor, cabozantinib

## Abstract

**Simple Summary:**

Renal cancer is within the top 10 most common malignancies worldwide, of which clear cell histology represents the most common subtype in this cancer. Within the past few years, immunotherapy has been approved as a first-line therapy for metastatic renal cell carcinoma. Immunotherapy is a highly effective treatment that enhances the immune system’s ability to attack tumor cells. However, there are a subset of patients whose cancer progresses while on immunotherapy. These patients are then treated with a clinical trial which involves new combinations and types of therapies. This review article aims to summarize the most current data regarding first-line treatments and ongoing clinical trials in the expanding treatment landscape for metastatic renal cell carcinomas.

**Abstract:**

Immune checkpoint inhibitors (ICI) are now the bedrock for the treatment of metastatic renal cell carcinoma (RCC). Clear cell RCC (ccRCC) represents the most common subtype of this malignancy. Herein, we explore the therapeutic landscape of ccRCC by discussing the standard of care whose backbone consists of immune checkpoint inhibitors (ICI) and vascular endothelial growth factor inhibitors (VEGF). For ccRCC, pembrolizumab-axitinib, pembrolizumab-lenvatinib, and avelumab-axitinib or nivolumab-cabozantinib are now FDA-approved frontline options for all risk groups while nivolumab-ipilimumab is reserved for intermediate- and poor-risk groups. Monotherapy with pembrolizumab or nivolumab is a potential option for patients who are unable to take VEGFR-tyrosine kinase inhibitors. While outcomes have improved with the adoption of ICI therapies, many patients develop therapy-resistant disease, creating an unmet need for further investigation. The efficacy of novel therapies as well as novel combinations in the post-ICI era is unclear. This review summarizes the most significant clinical trials involving dual ICI/ICI and ICI/VEGFR therapies, in addition to other selected combination therapies that are likely to inform management in the near future.

## 1. Introduction

Renal cell carcinoma (RCC) is among the top ten most common cancer diagnoses in the USA, with an estimated 76,000 new cases projected per year [1]. Approximately one third of these patients with RCC will present with metastases at diagnosis. Clear cell RCC (ccRCC) accounts for approximately 80% of all kidney cancers and historically has been associated with a poor prognosis in the metastatic setting [2,3]. Prior to 2005, there were few effective systemic treatment options for the management of RCC. The mainstays of treatment previously included cytokine-based therapies such as interferon-alpha (IFN-a) and high-dose interleukin-2 (IL-2). These therapies were associated with a poor overall response rate [4], as well as considerable toxicities [5].

Thankfully, the last two decades have witnessed remarkable progress in the management of RCC. An increased understanding of the oncogenesis of RCC has led to the development of several targeted treatment options, including tyrosine kinase inhibitors (TKIs), vascular endothelial growth factor (VEGF) targeted agents, and mammalian target of rapamycin (mTOR) inhibitors. Furthermore, immune checkpoint inhibitors (ICIs) have emerged as an effective treatment option, both as a monotherapy and in combination with these other agents, leading to marked improvements in clinical outcomes.

The emergence of immune-checkpoint inhibitor (ICI)-based therapies have transformed the treatment landscape for patients with mRCC. Data from multiple clinical trials examining ICIs either as a dual immunotherapy or in combination with anti-VEGF targeted agents demonstrate significantly improved overall survival (OS), progression free survival (PFS), and overall response rate (ORR), compared to sunitinib. These clinical trials have led to the FDA approval of multiple ICI-based combination regimens as first-line treatment options for RCC. The International Metastatic Renal Cell Carcinoma Database Consortium (IMDC) criteria classify RCC subclassified into favorable, intermediate, and poor prognosis groups [6]. The criteria that incorporate clinical and laboratory risk factors were initially validated in patients undergoing VEGF therapies yet now continue to be used to guide systemic treatment selection in the era of ICI-based therapies. Current guidelines from the European Association of Urology recommend dual immunotherapy (ICI/ICI) as a first-line treatment for IMDC intermediate- and poor-risk groups and recommend combined ICI/VEGF therapies as first-line treatments across all IMDC risk groups. While the treatment options for RCC have shown significant advancements in recent years, the 5-year relative survival rate remains low, at only 13% in distant stage mRCC [7].

In this review, we summarize the key clinical trials that have contributed to the approval of ICI-based combination therapies as first-line treatment options for advanced RCC. We also describe several current ongoing clinical trials, with a focus on Phase 3 trials where available, review data regarding emergent therapies, and explore the new advances in diagnostics to further describe the treatment and genomics of this malignancy. Topics outside the scope of this narrative review are the role of ICI in the perioperative RCC setting, the role of cytoreductive nephrectomy in advanced stage RCC, active surveillance in metastatic RCC, and the management of non-clear cell RCC. 

## 2. Current First Line Standard of Care

### 2.1. ICI/ICI Combination Therapy for Intermediate- and Poor-Risk Groups

**Figure 1:**
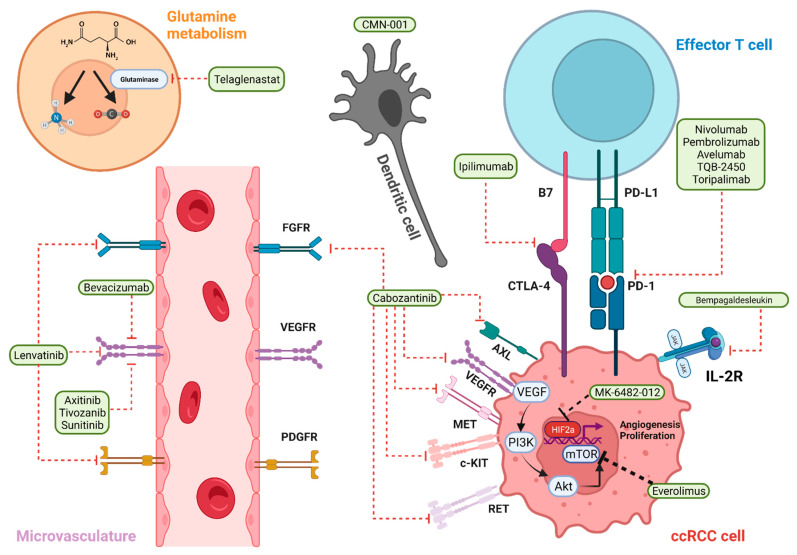
Current and novel therapies for mccRCC and their respective molecular targets. Created using Biorender^®^.

RCC is one of the most immune-infiltrated tumor types, thus, ICIs have been identified as a promising therapeutic option [8]. ccRCC has significant intratumor heterogeneity with known cases of negative PD-L1 that are responsive to ICI therapy. PD-L1 immunohistochemistry is not used in the IMDC risk stratification of mRCC. In fact, PD-L1 expression in mRCC is not a predictive biomarker for treatment selection, unlike many other tumor types [9,10]. ICIs have been shown to have efficacy as monotherapies or in combination with other agents, including other ICIs and VEGF-targeted agents (Figure 1). The ICIs currently used in the treatment of mRCC include agents that target the programmed cell death 1 (PD-1) receptor (nivolumab, pembrolizumab), programmed death ligand 1 (PD-L1) (e.g.,atezolizumab, avelumab, and durvalumab), and cytotoxic T lymphocyte antigen 4 (CTLA-4) (ipilimumab). Table 1 delineates the key findings from the most established clinical trials for ccRCC.

To date, the only approved dual ICI combination regimen is nivolumab plus ipilimumab, which has emerged as one of the major first-line treatment options for patients with intermediate- or poor risk- RCC based on data from the **CheckMate-214** trial [16]. The trial is a phase 3 study investigating the efficacy of nivolumab and ipilimumab (experimental arm) vs. sunitinib (control arm) in 1096 treatment-naïve patients with intermediate- or poor-prognostic risk advanced RCC. The co-primary endpoints for the trial were OS, ORR, and PFS. 

The OS and ORR were significantly improved with nivolumab-ipilimumab compared to sunitinib in the intermediate-poor risk group. The median follow-up was 25.2 months. The 18-month OS was 75% in nivolumab-ipilimumab compared to 60% in sunitinib. The median OS was not reached in the nivolumab-ipilimumab group versus 26.0 months in sunitinib. The ORR was 42% in nivolumab-ipilimumab compared to 27% in sunitinib. A trend was observed in favor of nivolumab-ipilimumab vs. sunitinib for PFS (11.6 vs. 8.4 months, respectively), although there was no statistical significance. These findings led to the FDA approval of this dual immunotherapy regimen in April 2018. 

Nivolumab-ipilimumab is one of the most effective ICI options with the caveat that treatment-related toxicities are common in the initial period of the treatment [17]. Although there were fewer grade 3–4 TRAEs in nivolumab-ipilimumab (48%) vs. sunitinib (64%), a high index of suspicion for immune-related adverse events is needed when patients are receiving both drugs in the first four cycles (before switching to maintenance nivolumab alone). Establishing itself as the longest phase 3 follow up for ICI combination therapy, an extended 5-year follow-up of CheckMate-214 continued to demonstrate the clinical benefits of nivolumab-ipilimumab over sunitinib. In an intention-to-treat (ITT) analysis, improved OS was sustained in nivolumab-ipilimumab compared to sunitinib in intermediate-poor risk groups [55.7 months vs. 38.4 months; HR 0.68 (95% CI: 0.58–0.81, *p* < 0.0001)] [11]. The conditional response in the ITT analysis was preserved beyond the 3-year point in 89% vs. 63% of patients on nivolumab-ipilimumab or sunitinib, respectively [18]. Thus, this dual immunotherapy regimen currently continues to serve as one of the main first-line treatment options for patients in IMDC intermediate- and poor-risk groups.

### 2.2. ICI/VEGF Combination Therapy Options for All Risk Groups

Nearly 90% of ccRCC tumors have a loss of heterozygosity at chromosome 3p, with a resulting loss of function of the pVHL tumor suppression protein (von Hippel-Lindau), leading to the activation of the hypoxia inducible factor-2-alpha (HIF-2α) transcription factor. HIF-2α is involved in angiogenesis, cell migration, and tumor proliferation. VEGF inhibitors and TKIs are efficacious in targeting the downstream effects of HIF-2α activation, leading to tumor response in RCC. Several ICI/VEGF combination therapy regimens have been approved as first-line therapy options for patients of all IMDC risk groups based on promising results from four major clinical trials: JAVELIN-101, Keynote 426, CheckMate 9ER, and CLEAR.

The **JAVELIN Renal 101** was the first trial to report on ICI/VEGF combination therapy for RCC [19]. JAVELIN-101 was a phase 3 trial that examined a combination of axitinib with avelumab compared to sunitinib in 886 treatment-naïve patients with advanced RCC across all IMDC risk groups. The primary endpoints included PFS and OS in patients with tumors positive for PD-L1 expression (PD-L1+). The overall response rate (ORR) was also assessed. PD-L1+ patients were found to make up 63.7% of the cohort and demonstrated a significantly greater PFS [(13.8 vs. 7.2 months; HR 0.61 (95% CI: 0.47–0.79, *p* < 0.001)] and ORR (55.2% vs. 25.5%) in axitinib-avelumab vs. sunitinib, respectively. In the cohort overall (irrespective of PD-L1 expressivity), PFS was also found to be higher in the axitinib-avelumab group compared to sunitinib [(13.8 vs. 8.4 months; HR 0.69 (95% CI: 0.56–0.84, *p* < 0.001)]. In terms of the safety profiles for these agents, TRAEs were similar between experimental and control groups (AEs occurred in 99.5% vs. 99.3%; AEs of grade 3 or higher occurred in 71.2% vs. 71.5%). The findings of the JAVELIN Renal 101 trial led to the FDA approval of the axitinib-avelumab combination regimen for all IMDC risk groups in May of 2019.

The follow-up data published in August 2020 from the JAVELIN-101 trial continued to show the therapeutic advantage of axitinib-avelumab over sunitinib with respect to PFS in both PD-L1+ patients and in the overall population [(**PD-L1+:** HR 0.62 (95% CI 0.490–0.777), *p* < 0.0001; median PFS: 13.8 months (95% CI 10.1–20.7) vs. 7.0 months (95% CI 5.7–9.6); **Overall population**: HR 0.69 (95% CI 0.574–0.825); *p* < 0.0001; median PFS: 13.3 months (95% CI 11.1–15.3) vs. 8.0 months (95% CI 6.7–9.8)]; however, the OS data were immature for all groups in the most recent analyses [20]. An updated analysis had enrolled 886 patients by 2021 and continued to show maintained efficacy consistent with prior studies [12]. The lack of demonstrated OS benefit continues to deter many clinicians from using this regimen at this time. According to the current European Association of Urology Guideline, updated in October 2021, this combination therapy is not recommended until a significant survival signal can be demonstrated [21].

**Keynote-426** was another phase 3 trial examining the efficacy of ICI/VEGF combination therapy [22]. This study comprised 861 patients of all IMDC risk groups, who were randomly assigned either to a combination of pembrolizumab and axitinib or to the control arm of sunitinib. The primary endpoints were OS and PFS in the ITT population. The ORR was also assessed. At a median follow-up of 12.8 months, pembrolizumab-axitinib was associated with significantly improved clinical outcomes compared to the sunitinib arm, including greater PFS [(15.1 months vs. 11.1 months; HR 0.69 (95% CI: 0.57–0.84, *p* < 0.001)], improved 12-month OS rate [(90% vs. 78.3%; HR: 0.53 (95% CI: 0.38–0.74; *p* < 0.0001)], and higher ORRs (59.3% vs. 35.7%). These outcomes were found across all IMDC groups, irrespective of tumor PD-L1 expression. The frequency of TRAEs were similar between the experimental and control arms, with TRAEs of grade 3 or higher observed in 75.8% of patients in pembrolizumab-axitinib and 70.6% in sunitinib. The results from this trial led to the FDA approval of pembrolizumab-axitinib for all IMDC risk groups in April 2019. Follow-up data in October 2020 showed a sustained clinical benefit in the pembrolizumab-axitinib group compared to sunitinib with respect to OS [(NR vs. 35.7 months; HR 0.68 (95% CI 0.55–0.85), *p* = 0.0003] and PFS (median 15.4 months vs. 11.1 months HR 0.71, *p* < 0.0001) [23]. An extended follow-up published in May 2021 further demonstrated the advantage of pembrolizumab-axitinib vs. sunitinib with respect to the 42-month OS rate (57.5% vs. 48.5%) and PFS rate (25.1% vs. 10.6% with sunitinib), and the ORR (60.4% vs. 39.6%) [13]. Of note, the 42-month OS benefit compared to sunitinib is smaller than expected (less than 10%) as the OS curves for each appear to be very close to the extended follow up. 

Another pivotal trial investigating the efficacy of ICI/VEGF combination therapy was **CheckMate 9ER**, a phase 3 study that assessed the efficacy of combination therapy with nivolumab and cabozantinib compared to sunitinib in previously untreated patients with advanced ccRCC [24]. The study comprised 651 patients, with the primary endpoint being PFS. The secondary endpoints included OS and ORR, and an exploratory endpoint of the health-related quality of life was also assessed. The dose of cabozantinib in the trial was 40 mg daily, which is less than the 60 mg daily dose when cabozantinib is used alone. At a median follow-up of 18.1 months, the median PFS was significantly higher in nivolumab-cabozantinib compared to sunitinib (16.6 months vs. 8.3 months) in all risk groups, regardless of their PD-L1 status. Nivolumab-cabozantinib also showed improved clinical outcomes compared to sunitinib with respect to the 12-month OS rate (85.7% vs. 75.6%) and ORR (55.7% vs. 27.1%). The safety profiles were similar between nivolumab-cabozantinib and sunitinib, with rates of TRAEs of grade 3 or higher observed in 75.3% and 70.6%, respectively. In the nivolumab-cabozantinib group, 19.7% discontinued at least one of the drugs, and 5.6% discontinued both. Overall, patients reported better health-related quality of life with nivolumab-cabozantinib compared to sunitinib. Based on these findings, the combination nivolumab-cabozantinib therapy received FDA approval for all IMDC risk groups in January 2021.

Updated results from the CheckMate 9ER study were published in March 2021 [14]. At a median follow-up of 23.5 months, nivolumab-cabozantinib continued to show a significant improvement in PFS compared to sunitinib [(17.0 months vs. 9.3 months; HR 0.52 (95% CI: 0.43–0.64, *p* < 0.0001)]. Nivolumab-cabozantinib was also advantageous to sunitinib with respect to OS [(NR vs. 29.5 months, HR: 0.66 (95% CI: 0.050–0.87, *p* = 0.0034)] and ORR (54.8% vs. 28.4%). 

Most recently, the results from the phase 3 **CLEAR** study compared pembrolizumab-lenvatinib (pem-len), an ICI/VEGF combination therapy, to either lenvatinib-everolimus (len-eve) or sunitinib in a 1:1:1 ratio [15]. The CLEAR trial examined a cohort of 1069 treatment-naïve patients with advanced RCC across all IMDC groups. The primary endpoint was PFS, with OS and ORR also assessed. The results indicated a PFS benefit with pem-len compared to sunitinib (HR 0.39, median PFS: 23.9 months versus 9.2 months, *p* < 0.001). Pem-len also showed an advantage over sunitinib with respect to OS [HR 0.66 (95% CI: 0.49–0.88, *p* = 0.005)] and ORR (71% in pem-len vs. 36.1% in sunitinib). These improved clinical outcomes were observed across all IMDC risk groups, irrespective of PD-L1 expressivity. The dose reductions for treatment related toxicity were common in the experimental arm (68.8% vs. 50.3%), with TRAEs of grade 3 or higher occurring in 82.4% and 71.8% in pem-len and sunitinib, respectively. Such a high incidence of grade 3 or higher TRAEs could be explained by the 20 mg/day lenvatinib dosing in the trial, which is higher than 18 mg/day when used in combination with everolimus. This also explains the frequent requirement for dose modification. Importantly, CLEAR had the highest ORR difference between the experimental arm and sunitinib, leading some investigators to propose that this could be a regimen of choice as it is most important to achieve a rapid disease response. 

The CLEAR study also demonstrated a statistically significant improvement in PFS in the pem-len group relative to the len-eve and sunitinib groups [HR 0.65 (95% CI: 0.53–0.80, *p* < 0.001); median PFS: 23.9 vs. 14.7 vs. 9.2 months)]. The OS was longer with pembrolizumab plus lenvatinib than with sunitinib [(HR 0.66 (95% CI: 0.49–0.88; *p* = 0.005)]. These results led to the FDA approval of pem-len as a first line treatment for advanced ccRCC in August 2021. On the other hand, lenvatinib-everolimus is not currently recommended as a first-line treatment for mRCC; however, it is frequently used as a subsequent therapy [25].

For patients who cannot tolerate PD-1 inhibitors, sunitinib, pazopanib, and tivozanib can be offered as alternatives to immunotherapy for all IMDC risk patients. Cabozantinib is another option that serves as a PD-1 inhibitor alternative, and is available for patients with IMDC intermediate- or poor risk disease [26]. Recent studies have moved towards evaluating the efficacy of cabozantinib in the real-world setting, reporting it as a commonly utilized VEGF therapy and increasingly used as 2 L therapy after dual ICI therapy [27,28,29,30].

### 2.3. Treatment Section in the First Line Setting

A sizeable minority of patients with mccRCC are unable to receive ICI-doublet or ICI-VEGF due to comorbid conditions. For patients who have a contraindication to ICI, sunitinib, pazopanib, and tivozanib can be offered as alternatives for all IMDC risk patients. Cabozantinib is also available for patients with IMDC intermediate- or poor risk disease [26]. Recent studies support the efficacy of cabozantinib in the real-world setting, reporting it as a commonly utilized VEGF therapy and that it is increasingly used as 2 L therapy after dual ICI therapy [27,28,29,30].

While ICI/VEGF are better than ICI/ICI for the treatment of mccRCC in patients with favorable risk groups, there is currently no consensus on whether ICI/ICI or ICI/VEGF shows the greatest therapeutic benefit for intermediate- or poor-risk disease groups, which make up approximately 75% of patients with advanced RCC. Further, clinicians also have a number of disease ICI/VEGF combinations to choose from with no head-to-head comparison [31].

The choice of therapy, ICI/ICI vs. ICI/VEGF, is determined by several factors, including the long-term survival, initial burden of disease, dosing, and metastatic involvement. Dual ICI is preferred in patients where the most desired outcome is long-term survival as previously noted in the discussion of the Checkmate-214 trial. However, more patients could achieve disease progression early on, especially those who might be highly symptomatic. ICI/VEGF has been increasingly evaluated in the real-world setting demonstrating both improvement in symptoms and a favorable efficacy. ICI/VEGF is preferred in patients with an initial high-burden of disease in terms of symptoms and metastatic sites. For instance, an organ-specific metastatic involvement analysis of patients receiving a combination of nivolumab-cabozantinib showed ≥30% tumor size reduction in the kidney (89% of patients), lung (76%), lymph nodes (88%), and liver (73%) [32]. The downsides of the ICI/VEGF approach include the less mature OS data compared to Checkmate 214 along with the potential for overlapping toxicities (such as diarrhea) that would be difficult to differentiate as indicative of ICI-related vs. VEGF-related.

The specific involved disease sites are also important in the initial treatment selection approach. A recent pivotal multi-institutional study demonstrated significantly improved intracranial activity and a tolerable safety profile with cabozantinib therapy [33]. A total of 88 patients were divided into two cohorts, cohort A containing 33 patients without concomitant brain-directed local therapy at cabozantinib initiation, and cohort B with 55 patients and receiving concomitant brain-directed local therapy. The extracranial response rate was 48% (95% CI: 31–66%) in cohort A vs. 38% (95% CI: 25–52%). The median OS was 15 months in cohort A (95% CI: 9.0–30.0 months) vs. 16 months (95% CI: 12.0–21.9 months) in cohort B. While those results require validation, they suggest that the use of cabozantinib can be started before brain-directed therapy.

Patient preferences may be influenced by several different variables, including treatment schedule, treatment-related toxicities, management options for these potential toxicities, and whether the approach would be covered by the patient’s insurance provider [34,35]. For instance, pembrolizumab can be given every 6 weeks as opposed to every 4 weeks with nivolumab therapy—making pembrolizumab a preferred drug for patients who face a distance barrier to accessing an infusion center. Of the TKIs, lenvatinib is the only medication that can be crushed and mixed with water. A thorough discussion with patients can help guide the treatment selection among the array of possible drug regimens. Lastly, clinicians can also accumulate experience using a specific regimen (e.g., the ICI/VEGF regimen of choice that is supported by data) to improve patients’ outcomes through their being well versed in the specifics of the used regimen. This is important as head-to-head comparisons of the frontline ICI/VEGF in the clinical trial setting are lacking, and such studies are unlikely to be conducted.

## 3. Ongoing Trials and Emerging Treatments in the Frontline Setting

The ongoing approaches to improving the clinical outcomes in the frontline include a further intensification of therapy upfront with ICI/ICI/VEGF combinations, sequential therapy approaches, and/or the use non-newer targets (Table 2). It is postulated that sequential therapy may inhibit clonal evolution, and as a result, reduce the possibility of recurrence or relapse. Of the TKIs, cabozantinib is the one primarily tested in sequential treatment with ICI therapy in Phase 3 trials.

### 3.1. Efficacy of Cabozantinib in Sequential ICI-Based Therapy

**COSMIC 313** is a Phase 3 trial investigating 840 previously untreated patients with advanced ccRCC with an IMDC of intermediate or poor risk [36]. The intervention arm consists of three therapies at two doses that are sequentially administered: cabozantinib dosed at 40 mg oral daily, nivolumab dosed at 3 mg/kg intravenous every 3 weeks for 4 doses, and ipilimumab dosed at 1 mg/kg intravenous every 3 weeks for 4 doses. This regimen is then followed by cabozantinib 40 mg daily and nivolumab 480 mg IV once monthly. The control arm consists of a cabozantinib-matched placebo and the same treatment regimen for nivolumab and ipilimumab as the experimental arm. The primary endpoint is PFS. Nivolumab will be administered for up to two years. No further survival data has been published yet.

Sequential triple therapy is also explored in the **PDIGREE** trial, a phase 3 adaptive randomized study developed to elucidate the role of TKIs in metastatic ccRCC [53]. It specifically investigates the efficacy of cabozantinib as a maintenance therapy after frontline dual ICI therapy. Up to 1046 patients are estimated to receive ICI therapy during the induction phase, comprising nivolumab 3 mg/kg and ipilimumab 1 mg/kg intravenously. At the 3-month mark, patients will then be divided into CR or non-CR groups with changes in their regimen. Those who achieve CR will undergo nivolumab 480 mg administered intravenously every 4 weeks. Those with progressive disease will receive cabozantinib 60 mg once daily. Lastly, if the patients are non-CR/non-PD, those patients will be randomized to nivolumab 480 mg every 4 weeks vs. cabozantinib 40 mg oral daily. The 3-year overall survival is the overall endpoint and is hypothesized to be 70% for nivolumab-cabozantinib compared to 60% for nivolumab alone. The preliminary data is highly anticipated [37].

### 3.2. Role of IL-2 Agonists in ICI-Based Therapies

IL-2 agonists were historically administered in patients with ccRCC who have a good performance status and possess intact organ function. Its mechanism of action is hypothesized to enhance the immune system via cytotoxic T and natural killer cell activation to combat the tumor microenvironment [54]. While high-dose IL-2 has been employed in the treatment landscape, it involved an unfavorable safety profile and only a small minority of patients achieved a response. In contrast, bempegaldesleukin is an IL-2 receptor pathway agonist with a tolerable safety profile recently studied in the **PIVOT-09 trial,** a Phase 3 open-label investigation [55]. The PIVOT-09 trial aimed to characterize the efficacy of IL-2 and ICI therapy in the experimental arm compared to ICI and a choice of a TKI in the control arm (i.e., sunitinib or cabozantinib) in the frontline setting. The 1:1 randomization of 0.006 mg/kg of bempegaldesleukin plus nivolumab 360 mg intravenously every 3 weeks versus TKI (sunitinib 50 mg 4 weeks on, 2 weeks off or 60 g cabozantinib orally once daily) was performed. The results of the study have not been published. Unfortunately, a press release on 14 April 2022, by bempegaldesleukin and nivolumab’s developers indicated that its development for all ongoing studies will be stopped. Therefore, this combination is not likely to have a role in the frontline setting. 

**PIVOT IO-011** (NCT04540705) is a two-part multi-center phase 1/2 study exploring the efficacy of an overlapping triplet regimen (IL-2/ICI/TKI) in advanced ccRCC disease [38]. The experimental arm consists of bempegaldesleukin plus nivolumab and TKI vs. nivolumab and TKI (either cabozantinib or axitinib). The patients in Part 1 will receive bempegaldesleukin and nivolumab and either cabozantinib or axitinib. There will be two different dosing regimens for each TKI which will determine their RP2D. The primary endpoints in Part 1 include safety profiles and dose-limiting toxicities. For Part 2, patients will then be randomized to receive either bempegaldesleukin/nivolumab/cabozantinib or nivolumab/cabozantinib and stratified by the IMDC score and nephrectomy status. The primary endpoints in Part 2 include ORR per RECIST 1.1 criteria; secondary endpoints include safety profile, OS, and PFS. This study is currently recruiting with a total duration of <2 years for bempegaldesleukin/nivo and cabozantinib maintenance, estimated to be completed by January 2026. At the time of manuscript writing, it not yet clear how the decision to halt bempegaldesleukin + nivolumab in the PIVOT-09 trial will impact the enrollment and subsequent success of PIVOT IO-011.

### 3.3. Efficacy of Novel HIF-2α Inhibitors in ICI-Based Therapies

As mentioned previously, most cases of ccRCC present with the biallelic inactivation of the pVHL gene. Normally, VHL serves to ubiquitinate various transcription factors, notably the HIF protein family, allowing for proteasome-mediated degradation and the inhibition of tumorigenesis. However, when there is a mutational functional loss, HIF-2α, a tumorigenic transcription factor, travels to the nucleus and drives oncogenic gene expression [56,57]. Thus, there is an unmet need to further characterize the role of HIF2α inhibitors in the landscape of metastatic ccRCC therapy.

There have been several in vitro studies demonstrating that a functional delivery of HIF2α inhibitors can potentially silence genes and consequently slow tumor growth in xenografts. Belzutifan is a new therapy developed to selectively target HIF2α and subsequently inhibit hypoxia-driven tumorigenesis. **MK-6482-012** is an ongoing Phase 3 trial investigating the role of HIF2α inhibitors in the developing therapeutic landscape in ccRCC [58]. This trial compares three arms amongst each other: one containing the HIF2α inhibitor and ICI and the others containing ICI and VEGF inhibitors. The experimental arms specifically comprise belzutifan, pembrolizumab, and lenvatinib. The other treatment arms consist of ICI and VEGF therapy, specifically pembrolizumab, quavonlimab, and lenvatinib or pembrolizumab/lenvatinib. This study hypothesizes that the triple therapy containing a HIF2α inhibitor will demonstrate superior PFS and OS outcomes compared to dual ICI and VEGF therapy. The primary endpoint is PFS and OS.

An updated analysis in 2021 enrolled 55 patients and demonstrated that belzutifan has a tolerable side effect profile with only 4% (*n* = 2) of patients discontinuing therapy due to AEs. The ORR was 25% with 14 patients confirmed to develop PR, and 77% patients had a response by 6 months. 55% (*n* = 30) had SD. The mPFS was 14.5 months and the median duration of response (DoR) was not reached [41].

## 4. Subsequent Line Therapies

Treatment beyond frontline ICI/ICI or ICI/VEGF is an area of need in mRCC. While several therapies are emerging, the disease has the potential for an aggressive course and is frequently lethal. We highlight in this section both standard of care options and emerging therapies for the second-line and beyond, with a focus on patients who have already received ICI in the frontline setting.

### 4.1. Single-Agent TKI after ICI Therapy as Standard of Care

Single-agent TKI is the preferred approach after progression in an ICI-based frontline therapy setting within the NCCN guidelines. The guidelines support using a TKI that was not received as part of the frontline therapy, with cabozantinib and combination lenvatinib-everolimus being both Category 1 recommendations for clear cell histology. In fact, a meta-analysis had shown that cabozantinib was associated with a higher probability of longer OS and PFS as subsequent line therapy compared to everolimus, nivolumab, axitinib, sorafenib and best supportive care [59]. The use of lenvatinib-everolimus as a subsequent line of therapy is supported by the results of a Phase 2 study showing significantly better OS and PFS compared to everolimus alone in patients with advanced ccRCC [60]. It is worthwhile to note that in both studies patients received prior VEGFR inhibitors and not ICI as the studies predate the exponential use of ICI in frontline ccRCC treatment. 

Tivozanib (TIVO-3) is also now FDA approved in patients with relapsed or refractory RCC after two or more lines of therapy based on the results of the Phase 3 TIVO-3 trial. The TIVO-3 clinical trial evaluated the efficacy of VEGF treatment in those with diseases refractory to prior ICI therapy. The approval was based on the TIVO-3 trial involving 350 patients with ITT analysis. PFS improved with tivozanib versus sorafenib therapy [(5.6 months vs. 3.6 months, HR 0.73 (95% CI: 0.56–0.94; *p* = 0.016)], however, without a significant OS benefit. The response rate was 18% in the tivozanib arm vs. 8% with sorafenib. Fewer adverse events were reported in the tivonazib arm (84%) compared to 95% [46].

### 4.2. Data on ICI Re-Challenge after ICI Therapy

Multiple studies explore the efficacy of ICI re-challenge in ccRCC refractory to initial ICI therapy. Of those, we will discuss Ravi et al., HCRN GU 16-260, TITAN-RCC trials, and OMNIVORE.

In a multicenter retrospective cohort study, Ravi et al. explored the efficacy of ICI rechallenge in patients with mRCC [61]. The study included 69 patients between 2012–2019 who had at least two separate lines of ICI (named ICI-1 and ICI-2, alone or in combination with other therapies). The ORR was the primary endpoint. The ORR for ICI retreatment after ICI-1 was 37% and 23% at ICI-2. The greatest response was at ICI-2 among those who previously responded to ICI-1. The study suggested that retreatment might re-sensitize patients to subsequent ICI while acknowledging the limitations related to the retrospective nature, encouraging further prospective studies.

**TITAN-RCC** evaluated 258 patients by dividing them into two groups: those who received ICI either first line or second line in setting of receiving prior TKI therapy [62]. The primary endpoint was ORR. All patients were started with nivolumab 240 mg biweekly. Those with CR or PR were continued on monotherapy but were eligible to receive subsequent nivolumab therapy if they progressed or were stable by week 16. An updated analysis revealed an ORR of 28% in nivolumab monotherapy for the first line treatment group and 17% for second line [49]. In the first line group with an initial progressive disease, PFS was 6.3 months [(95% CI 3.7–10.1) in first line vs. 3.7 months (95% CI 2.0–4.5) in second line]. The OS was 27.2 months (95% CI 19.9-NR) in first line and 20.2 months (95% CI: 15.6-NR) in second line. While nivolumab monotherapy did demonstrate some efficacy, with recent advancements, patients are now only rarely treated with frontline nivolumab alone. The combination treatments as described above are more favored for eligible patients. 

**HCRN GU 16-260** investigated the role of salvage nivolumab monotherapy and nivolumab and ipilimumab in 123 patients with ccRCC who progressed on initial nivolumab therapy [63]. The primary endpoints were the ORR and mPFS. This regimen demonstrated an ORR of 29.3%, however, 30.7% progressed upon therapy. The mPFS was 7.4 months but 28 patients were unable to continue with the study due to progressed disease or TRAEs. Grade 3-5 TRAEs were significant while on ICI. This data demonstrates salvage therapy with nivolumab and ipilimumab after nivolumab monotherapy was not feasible from both a response rate and safety profile context. Thus, this regimen is no longer commonly utilized.

**OMNIVORE** is a Phase 2 response adaptive trial that enlisted patients with any RCC histology without prior ICI treatment [47]. All patients received induction nivolumab either dosed at 240 mg every 2 weeks or 480 mg every 4 weeks for a total course of at least 8 weeks. If SD or PD was achieved, then ipilimumab was added. Of the 83 treated patients, 57 had either SD or PD on nivolumab. Only two patients (4%) converted to partial response when ipilimumab was added, with no complete responders. Given this low conversion rate, the strategy of single-agent nivolumab followed by response-based ipilimumab is not recommended. It is also a proof of concept that re-treatment with ICI after ICI-progression is not likely to result in significantly improved clinical outcomes.

### 4.3. ICI/VEGF Therapy after ICI Therapy—Published and Ongoing

**KEYNOTE-146** is a phase 1b/2 study that investigated lenvatinib with concurrent ICI therapy in those who initially progressed with ICI [43]. A total of 104 patients with ccRCC were enrolled and were divided into three groups: (1) treatment-naïve patients who received the study drugs as first line, (2) patients with non-ICI based prior therapy, and (3) those previously treated with ICI. The ORR at week 24 was the primary endpoint and was 72% in treatment-naïve patients, 41% in non-ICI pre-treated, and 55.8% in ICI pre-treated. As for other measures, the mPFS for treatment-naïve patients was 24.1 months, for non-ICI pre-treated 11.8 months, and for ICI pre-treated 12.2 months. The median OS was 30.3 months in non-ICI pre-treated, and it was not reached in both treatment-naïve and ICI-pre-treated patients. This regimen is therefore considered to be an option as a subsequent line of therapy. 

**CONTACT-03** is a trial investigating the combination of VEGF inhibitors and ICI in patients with progression on initial ICI therapy [44]. This trial hypothesizes that atezolizumab, when coupled with anti-angiogenic therapy, has utility in treating refractory metastatic RCC. It is a multicenter study seeking to enroll around 500 patients to compare cabozantinib and atezolizumab vs. cabozantinib as second-/third-line therapy in progressed RCC in setting of prior ICI therapy. The trial completed enrollment in January 2022. The results are currently pending.

**TiNIVO-2** (NCT04987203) is an ongoing phase 3 randomized, multicenter, controlled, and open-label study comparing tivozanib in combination with nivolumab against tivozanib monotherapy in patients with advanced ccRCC progressed on at least one line of therapy, including ICI [45]. An estimated number of 326 patients are planned for an adequate ability to detect a 50% improvement in PFS (12 vs. 8 months). The primary objective will be PFS, and the secondary endpoints include OS, ORR, and DoR. The patients will be stratified into two arms based on IMDC risk score and whether ICI was given in the most recent treatment line. Both arms will receive tivozanib 1.34 mg for 3 weeks followed by a week off. The combination arm will receive nivolumab 480 mg every 4 weeks. The serial follow-ups include imaging every 8 weeks for 2 years following C1D1 and every 3 months subsequently until disease progression is determined.

### 4.4. HIF-2α/VEGF Therapy after ICI Therapy

An ongoing Phase 2 trial (NCT03634540) investigates belzutifan plus cabozantinib for patients with advanced ccRCC who either are treatment naïve (cohort 1) or have previously received ICI (cohort 2). In a recent update of 52 patients enrolled on cohort 2, 28 (54%) had received one prior line of therapy and 24 (46%) had received two prior lines of therapy. The ORR was 28.8% for all IMDC risk categories and in patients who received prior line of therapies with no new safety signals [64]. These results highlight that belzutifan plus cabozantinib may prove to be a promising combination. The final results are needed before this combination can be used in clinics outside of a clinical trial.

### 4.5. Additional Targeted Pathways with Mixed Results

Many studies, both in vivo and in vitro, have demonstrated glutamine’s role in promoting tumorigenesis. This pathway was thoroughly investigated as a therapeutic target in advanced ccRCC [65]. As a review, glutaminase converts glutamine to glutamate which acts as a building block for TCA intermediates to fuel lipogenesis, protein synthesis, and glucose metabolism, driving tumor growth [66]. Therefore, the overexpression of glutaminase contributes significantly to tumorigenesis. Several studies have demonstrated that constitutive HIF-2α action enhances glutamine metabolism. Of note, VHL mutation is a major genetic driver in ccRCC tumor growth. Since a VHL mutation results in unchecked HIF-2α function, glutamine metabolism may be accelerated, promoting growth within the tumor microenvironment [67]. Glutamine metabolism is also known to fuel tumorigenesis in hereditary leiomyomatosis and renal cell cancer (HLRCC), further highlighting this pathway as a pathogenic contributor.

**ENTRATA** explored the efficacy of telaglenastat, a glutaminase inhibitor with an mTOR inhibitor in RCC [50]. A total of 69 patients (61 of which had received PD-1/PD-L1 therapy) were randomized to either receive telaglenastat with everolimus or a placebo with everolimus until PD or the development of significant TRAEs. The primary endpoint was mPFS. The mPFS was 3.8 months for the group receiving telaglenastat with everolimus vs. 1.9 mo in placebo with everolimus group. There were no TRAE-related deaths.

Subsequently, the enthusiasm regarding telaglenastat faded with the negative results of **CANTATA**. This was a randomized phase 2 trial that evaluated the efficacy of telaglenastat in combination with cabozantinib versus a placebo plus cabozantinib in 444 patients post-ICI [51]. The mPFS was 9.2 mo in telaglenastat and cabozantinib vs. 9.3 months for the placebo-containing regimen (95% CI: 0.74–1.21; *p* = 0.65). The ORR was 31% in telaglenastat and cabozantinib vs. 28% in placebo and cabozantinib. The OS was not reached. Despite in vitro studies revealing a possible theoretical synergy with telaglenastat and cabozantinib in the tumor microenvironment, this trial demonstrates this combination demonstrates no substantially improved efficacy. 

There are also several other phase 2/3 randomized clinical trials currently under investigation which are further discussed in Table 2. Of those, dendritic cell immunotherapy is emerging as an exciting new treatment. A phase II trial is investigating the efficacy of CMN-001, an autologous dendritic cell therapy, combined with a dual ICI blockade with an accrual estimated to be completed by this spring [52]. Toripalimab/axitinib and TQB2450 (an experimental injection)/anlotinib are also new treatments being evaluated in comparison to sunitinib and may also show promise in fitting themselves into the treatment landscape, with an accrual estimated to be completed in the near future [39,40].

## 5. Conclusions

In summary, the therapeutic options for the treatment of mRCC continue to rapidly evolve, demonstrating marked improvements in response rates and survival for this aggressive and historically difficult-to-treat disease. The current first-line treatment options include dual immunotherapy (ICI/ICI) for intermediate-or -poor prognostic risk groups, or ICI/VEGF combination therapy for all risk groups. There are no currently validated biomarkers for the treatment selections. While those combinations achieved improvement in OS, not all patients achieved a long-term response. Ongoing clinical trials are currently examining the synergistic potential of additional therapies combining ICI, VEGF, and HIF1-α agents, as well as investigating the timing of these agents both for first and subsequent lines of therapy. The ongoing trials highlighted in this review are likely to provide more insight into improving outcomes in mRCC. 

## Figures and Tables

**Table 1:** Tabulation of first-line therapies in metastatic renal cell carcinoma with initial and follow-up results.

Trial Design						Initial Results				*Extended Follow-Up*
Trial	NCT	# Pts	Experimental Arm	Control Arm	Primary Endpoints	ORR%	PFS (mo)	OS (mo)	FDA approval	*Key Results*
CheckMate-214 [11]	02231749	1096	Nivolumab + Ipilimumab	Sunitinib	OS, ORR, PFS	42% vs. 27%	11.6 vs. 8.4	NR vs. 26.0	16 April 2018	OS: 55.7 vs. 38.4 mo PFS: 12.3 vs. 12.3 mo ORR: 39.3% vs. 32.4%
JAVELIN Renal 101 [12]	02684006	886	Axitinib + Avelumab	Sunitinib	OS (in PDL1+), PFS	55.2% vs. 25.5%	13.8 vs. 8.4	13.8 vs. 8.4	25 May 2019	PFS: 13.8 mo vs. 7.0 mo
Keynote-426 [13]	02853331	861	Pembrolizumab + Axitinib	Sunitinib	OS, PFS	59.3% vs. 35.7%	15.1 vs. 11.1	57.5% vs. 48.5 (rate)	19 April 2019	PFS rate: 25.1% vs. 10.6%; ORR: 60.4% vs. 39.6
CheckMate-9ER [14]	03141177	651	Nivolumab + Cabozantinib	Sunitinib	PFS	55.7% vs. 27.1%	16.6 vs. 8.3	NR vs. 29.5	22 January 2021	OS: NR vs. 29.5 mo PFS: 17.0 mo vs. 9.3 mo ORR: 54.8% vs. 28.4%
CLEAR [15]	02811861	1069	Pembrolizumab + Lenvatinib	Sunitinib	PFS	71% vs. 36.1%	23.9 vs. 9.2	33.6 vs. NR	10 August 2021	N/A

Abbreviations: # = number, ORR = overall response rate; PFS = progression-free survival; OS = overall survival, NR = not reached and TrAEs = treatment-related adverse events; PD-1 = programmed cell death protein-1; PD-L1 = programmed death-ligand-1 1.

**Table 2:** Tabulation of ongoing therapies in metastatic renal cell carcinoma.

Trial	Phase	NCT	# Pts	Experimental Arm	Control Arm	Primary Endpoints	Associated Measures (Accrual Date)
COSMIC 313 [36]	3	03937219	840	Cabozantinib + Nivolumab + Ipilimumab THEN Nivolumab + Cabozantinib	Cabozantinib + Nivolumab/ Ipilimumab + Placebo	PFS	Pending (March 2025)
PDIGREE [37]	3	03793166	1046	Nivolumab + Ipilimumab, + Cabozantinib for progression	Nivolumab	OS	Pending (September 2022) 70% vs. 60% (est)
PIVOT-011 [38]	1/2	03729245	251 (est)	Bempagaldeslukin + Nivolumab+ Cabozantinib	Nivolumab + Cabozantinib	ORR, dose toxicity, ORR	Pending (June 2024)
Toripalimab + axitinib [39]	3	04394975	380 (est)	Toripalimab + Axitinib	Sunitinib	PFS	Pending (June 2023)
TQB2450 [40]	3	04523272	418 (est)	TQB2450 + Anlotinib	Sunitinib	PFS	Pending (June 2023)
MK-6482-012 [41]	3	04195750	1431 (est)	HIF2α + ICI + VEGFRi	ICI + VEGFRi	TRAEs	Pending (September 2025)
Immotion-151 [42]	3	02420821	915	As above	As above	PFS, OS	11.2 mo vs. 7.2 mo
KEYNOTE-146 [43]	1b/2	02501096	104	Lenvantinib + ICI	None	ORR	72%, 41%, 55.8% (treated, non-ICI pre-treated, and ICI pre-treated)
CONTACT-03 [44]	3	04338269	500 (est)	Atezolizumab + Cabozantinib	Cabozantinib	PFS, OS	Pending (September 2022)
TiNIVO-2 [45]	3	04987203	326 (est)	Tivozanib + Nivolumab	Tivozanib	ORR	Pending (July 2024)
TIVO-3 [46]	3	02627963	350	Tivozanib	Sorafenib	PFS	5.6 mo vs. 3.6 mo
OMNIVORE [47]	2	03203473	83	Nivolumab +/− Ipililumab	None	PR or CR	14%
HCRN-GU16-260 [48]	2	03117309	123	Nivolumab THEN Nivolumab + Ipilimumab	None	ORR, mPFS	14.3%, 4.0 mo
TITAN RCC [49]	2	02917772	258	Nivolumab THEN Nivolumab + Ipililumab	None	ORR	37% (1st line), 28% (2nd line)
ENTRATA [50]	2	03163667	69	Telaglenastat + Everolimus	Everolimus + Placebo	PFS	3.8 mo vs. 1.9 mo
CANTRATA [51]	2	034288217	444	Telaglenastat + Cabozantinib	Cabozantinib + Placebo	PFS, OS	9.2 vs. 9.3 mo
Dendritic-cell immunotherapy [52]	2	04203901	120 (est)	CMN-001 + Nivolumab + Ipilimumab	Nivolumab + Ipililumab	OS	Pending (March 2022)

Abbreviations: # = number.
